# Trueness of digital implant impressions based on implant angulation and scan body materials

**DOI:** 10.1038/s41598-021-01442-9

**Published:** 2021-11-08

**Authors:** Jae-Hyun Lee, Jae-Hwi Bae, Su Young Lee

**Affiliations:** 1 Department of Prosthodontics and Dental Research Institute, School of Dentistry, Seoul National University, Seoul, 03080 South Korea; 2 Department of Prosthodontics, College of Medicine, Seoul St. Mary’s Hospital, The Catholic University of Korea, Seoul, 06591 South Korea

**Keywords:** Health care, Dentistry

## Abstract

Effects of implant angulation on digital implant impression accuracy remain controversial. Therefore, this in vitro study aimed to compare the digital implant impression trueness among models with different implant angulations and scan body materials. Six partially edentulous mandibular models with dental implants on the right second premolar and second molar areas were categorized according to the implant angulation of the distal implant (parallel, or 15° mesially or lingually tilted compared to the mesial implant) and scan body materials (polyetheretherketone or titanium). After scanning each model with intraoral scanners, the root mean square and within-tolerance values were calculated with respect to the reference, and nonparametric statistical tests were performed (α = .05). Scan data from models with the mesially tilted distal implant showed better trueness than the corresponding parallel and lingually tilted groups in terms of root mean square values (*p* < .017). The root mean square value in the titanium scan body group was lower than that in the polyetheretherketone scan body group (*p* < .001). However, the percentage within a tolerance of ± .1 mm was higher in the polyetheretherketone scan body group than in the titanium scan body group (*p* = .001). Intraoral scan data of models where the terminal implant was mesially tilted showed better trueness.

## Introduction

Intraoral scanners are used to obtain digital impressions since the introduction of computer-aided design and manufacturing systems for dental restorations^1^. Recently, digital implant impressions obtained using intraoral scanners have been widely used for the fabrication of dental implant restorations^1,2^. Compared to conventional implant impressions, digital implant impressions have advantages such as reduced impression-making time and elimination of patient discomfort due to the impression material, absence of volume changes in the impression, and ease of disinfection^1–3^. In addition, digital impressions enable more efficient model storage than conventional methods^1–3^.

Accurate fabrication of implant restorations requires an accurate implant impression. Digital implant impressions require using a scan body mounted on the implant. The acquired scan data of the scan body allow duplication of the implant position and angulation^4^. Thus, the scan body should be scanned accurately to obtain accurate digital implant impressions. In this regard, the following factors have been reported to affect their accuracy: implant location, angulation, and depth^5–8^; and scan body shape^4,9,10^.

Previous studies showed a favorable accuracy of conventional implant impressions when the implants were parallel^11,12^. This is because less deformation occurs when the tray is removed from parallelly placed implants. However, the effect of implant angulation on digital implant impression accuracy remains controversial. Some studies have reported better scan data accuracy in inclined implants than in parallel implants^5,13^. Another study reported worse accuracy in inclined implants^14^, and others have reported no significant difference^6,7,15–18^. Furthermore, the scan body design can affect the accuracy of digital implant impressions^10,19–21^. Various types of scan bodies developed by different manufacturers are being used, and recently, multi-purpose scan bodies made of biocompatible titanium have been introduced as a healing abutment^4^. However, scanning titanium scan bodies would be difficult to use in intraoral scanners because of their light-reflective surfaces. Therefore, this study aimed to perform a comparative evaluation of the trueness of digital impressions of multiple implants with various angulations obtained using polyetheretherketone (PEEK) and titanium scan bodies. The null hypothesis was that the implant angulation and scan body material would not affect the trueness of digital implant impressions.

## Materials and methods

**Figure 1:**
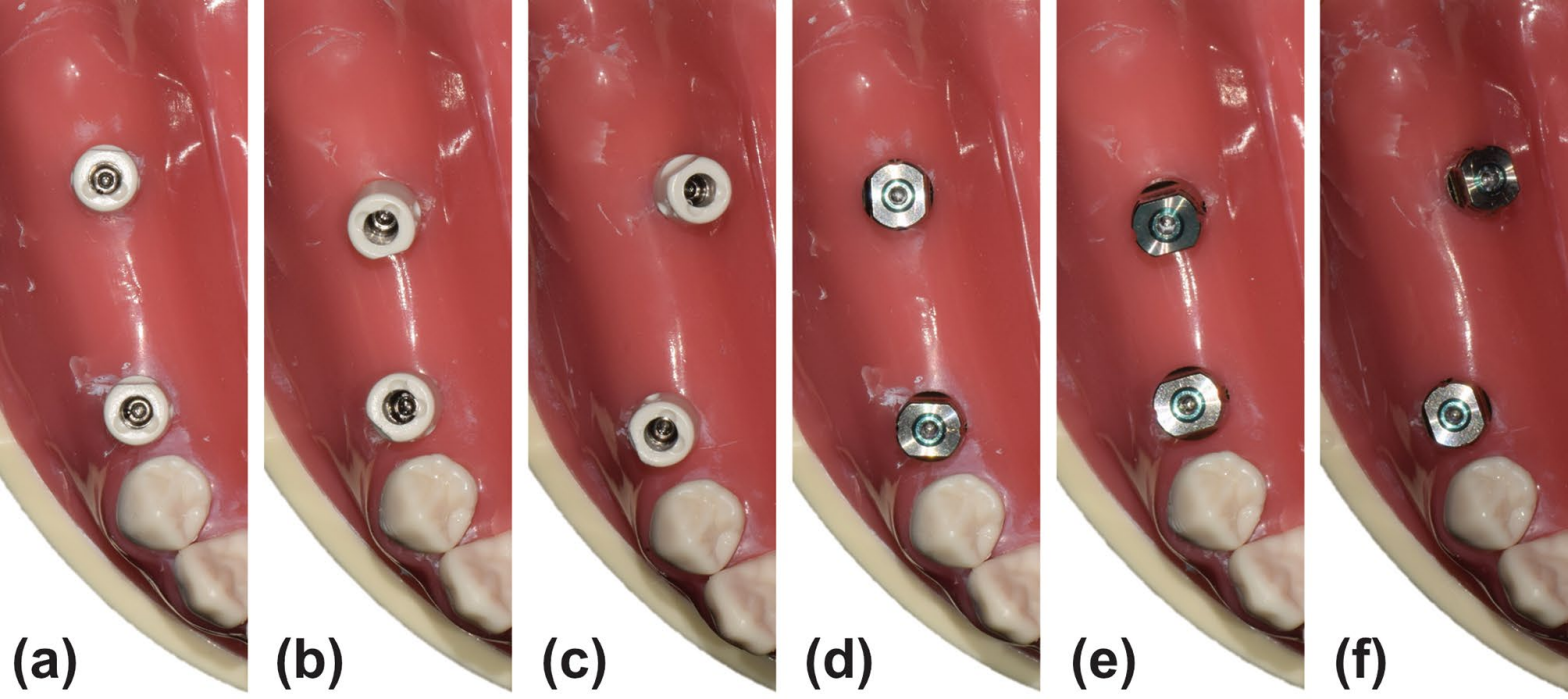
Study groups based on implant angulation and scan body materials. (**a**) Parallel implants with polyetheretherketone (PEEK) scan bodies. (**b**) Vertically placed mesial implant and 15° mesially tilted distal implant with PEEK scan bodies. (**c**) Vertically placed mesial implant and 15° lingually tilted distal implant with PEEK scan bodies. (**d**) Parallel implants with titanium scan bodies. (**e**) Vertically placed mesial implant and 15° mesially tilted distal implant with titanium scan bodies. (**f**) Vertically placed mesial implant and 15° lingually tilted distal implant with titanium scan bodies.

**Figure 2:**
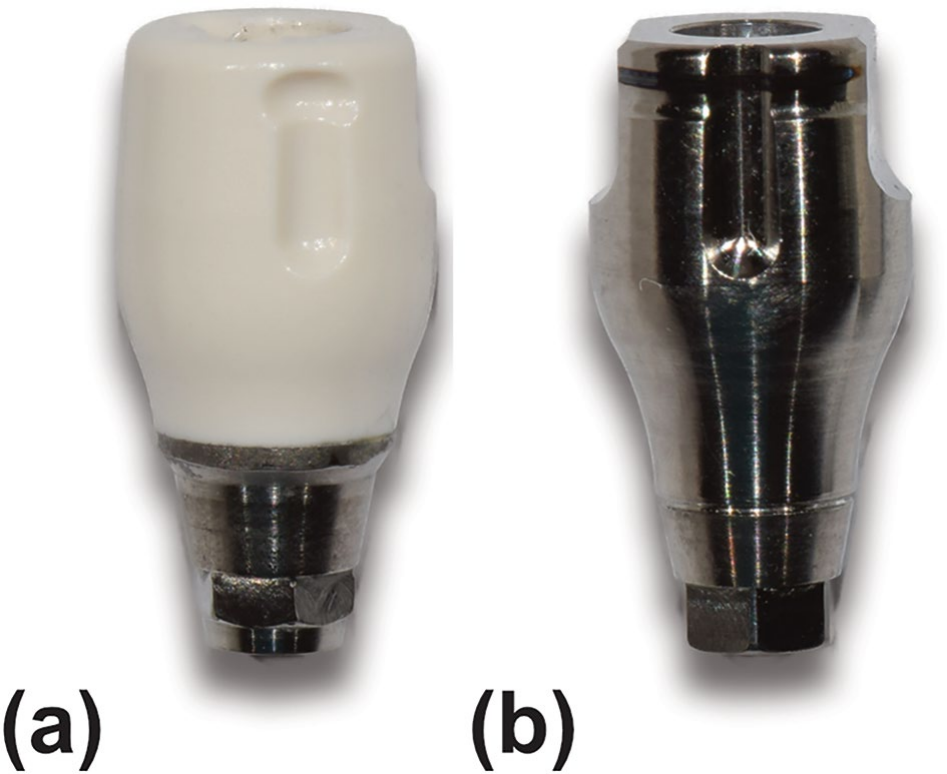
Scan bodies used in this study. (**a**) Polyetheretherketone scan body. (**b**) Titanium scan body.

Three mandibular models (DENTAL MODEL, M. Tech. Korea. Co., Gyeonggi-do, South Korea) with simulated gums made of elastic material and missing teeth on the right second premolar, first molar, and second molar areas were used in this study. Two internal connection-type bone-level implants (IS-III active, Neobiotech Co., Seoul, Korea) were placed in each of the three models at different angles using a surgical guide at the right second premolar and second molar regions. The angles between the two implants were as follows in the three models: the two implants were parallel, the distal implant was tilted 15° mesially to the mesial implant, and the distal implant was tilted 15° lingually compared to the mesial implant. In total, six groups were categorized by connecting either PEEK or titanium scan bodies (Myfit, Daegu, South Korea) on the three mandibular models (Fig. 1). The PEEK and titanium scan bodies were produced by the same manufacturer and had a similar shape, and their dimensions were approximately 8 mm in length (excluding the implant connection area) and 5.5 mm in diameter (Fig. 2).

**Figure 3:**
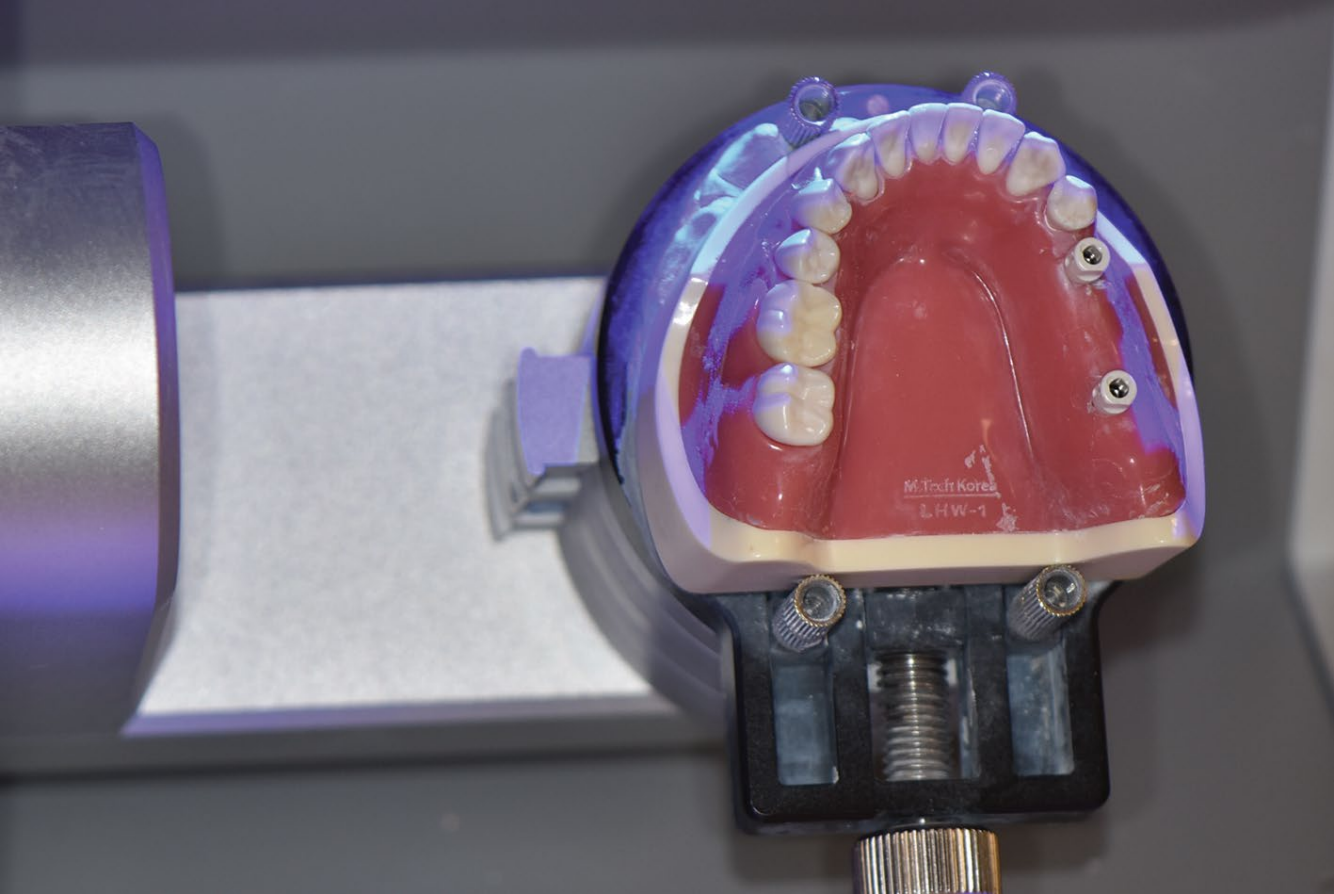
Creation of the reference scan data. Scanning of the models in each group with a desktop scanner (accuracy: 7 μm).

The models in the six groups were scanned using a desktop scanner (Identica T500; Medit Inc., Seoul, South Korea), with an accuracy (ISO 12836) of 7 μm (Fig. 3). Unnecessary scan areas were removed except for the scan body part by using a 3-dimensional modeling program (DentalCAD, exocad GmbH, Germany). The six scan data from each group were exported into the Standard Tessellation Language (STL) file format, which were used as reference scan data.

Three different intraoral scanners (CS3600, Carestream Dental; TRIOS3, 3shape; Primescan, Sirona Dental Systems) were used to obtain the scan data of the six groups. All intraoral scanning procedures were performed in the clinic, where the color temperature of the lights was 3900 K and the brightness was 400–500 lx. All scans were performed by a single experienced clinician. The occlusal plane of the model was maintained parallel to the floor, and the scanning procedure conducted as similar as possible to the clinical situation. The complete arch of the partially edentulous mandibular model was scanned. Intraoral scanning was performed 10 times for each group using each intraoral scanner. The resulting 180 scan datasets were obtained and exported in STL file format.

**Figure 4:**
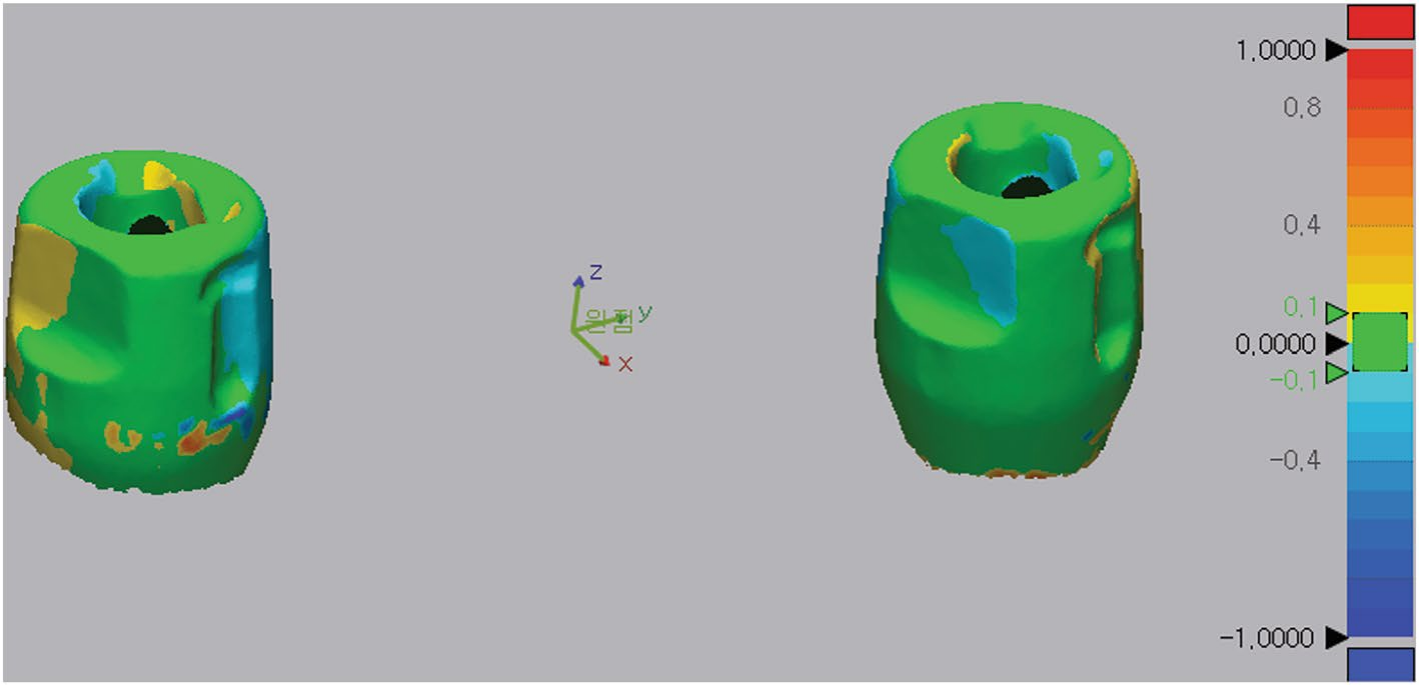
Three-dimensional (3D) deviation analysis. Colorimetric map of the 3D deviation.

Scan data accuracy was analyzed using 3D metrology software (Geomagic Control X, Geomagic, Rock Hill, South Carolina, USA). The reference and experimental scan data were superimposed and aligned using the best-fit alignment algorithm, and the deviation of the experimental scan data from the reference data was analyzed (Fig. 4). The software automatically calculated the root mean square (RMS) and within-tolerance values. RMS values were used to evaluate the trueness of the scan data obtained by intraoral scanners. The formula for calculating the RMS value is as follows:

$$\mathrm{RMS }=\sqrt{\frac{{\Sigma }_{\mathrm{n}=1}^{\mathrm{N}}{\left({\mathrm{x}}_{1,\mathrm{n}}-{\mathrm{x}}_{2,\mathrm{n}}\right)}^{2}}{\mathrm{N}}}$$

where x_1,n_ is a specific point in the reference STL file, x_2,n_ is a specific point in the experimental STL file, and N denotes the number of total corresponding points. In addition, the tolerance of the deviation values in the software was set to ± 100 μm based on a previous study^22^, and the ratio of the deviation values to be included within the tolerance was calculated and presented as %.

The data were analyzed using the statistical analysis software (SPSS Statistics 25.0, SPSS Inc., Chicago, IL, USA). Normal distribution was tested using the Shapiro–Wilk test. The results revealed that the data were not normally distributed. Therefore, data were analyzed using the Kruskal–Wallis test with a significance level of .05, followed by pairwise Mann–Whitney U tests. The significance level was adjusted using Bonferroni correction for multiple pairwise comparisons.

## Results

**Figure 5:**
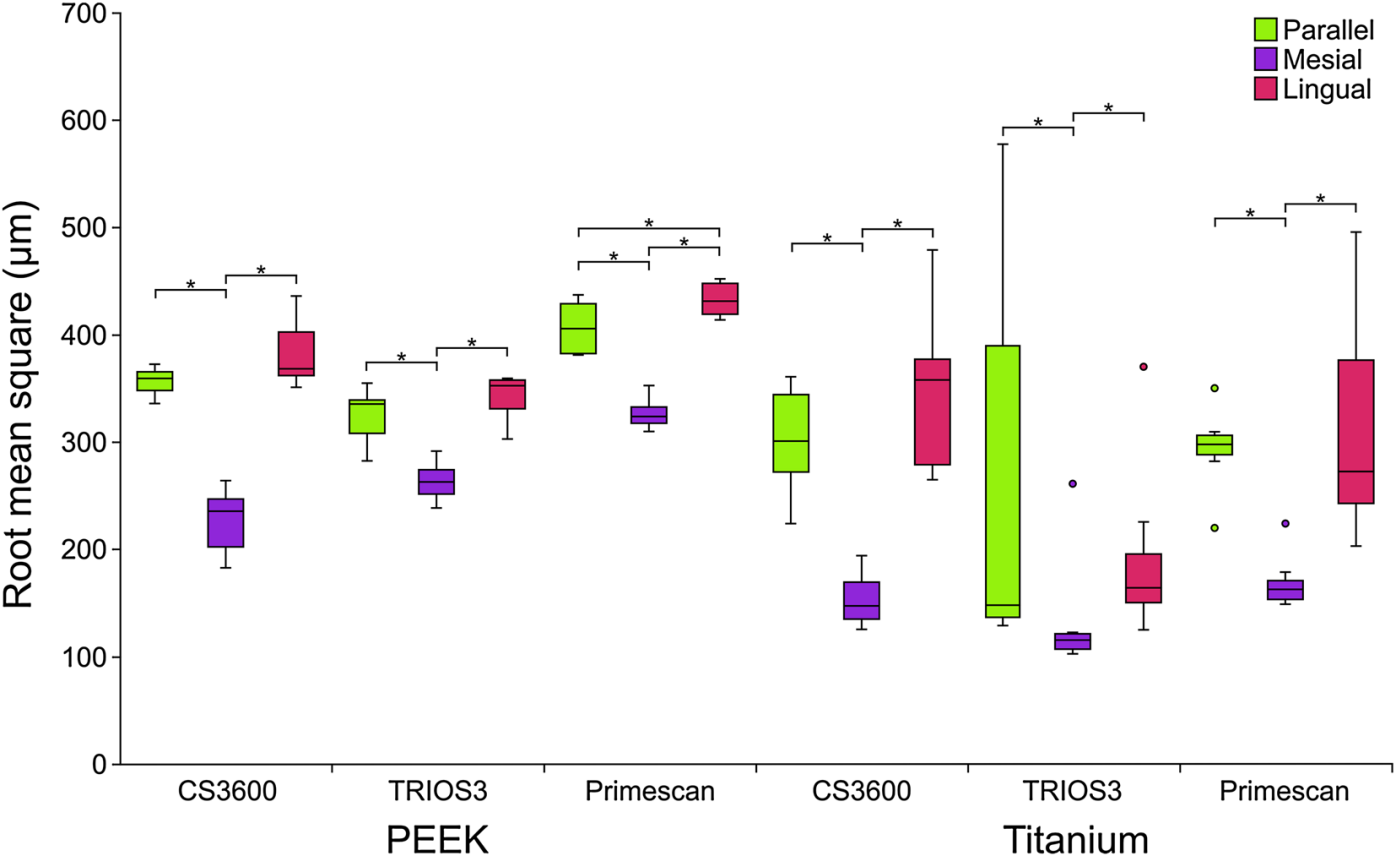
Trueness measurements in the two groups according to implant angulation, scan body materials, and intraoral scanners in terms of root mean square (RMS) 3-dimensional deviation. *Significant difference (Mann–Whitney U test followed by Bonferroni correction for multiple comparisons). *PEEK* polyetheretherketone.

The distribution of scan data trueness according to distal implant angulation, scan body materials, and intraoral scanners in terms of RMS values is shown in Fig. 5. The median (interquartile range) RMS for the mesially tilted distal implants with PEEK scan bodies were 235.6 (44.4), 263.1 (22.5), and 323.9 (15.1) µm, in the CS3600, TRIOS3, and Primescan scanner order, respectively. In addition, the groups with the titanium scan bodies and the mesially-tilted distal implant showed 147.5 (34.6) µm for CS3600, 115.5 (14.4) µm for TRIOS3, and 162.6 (17.6) µm for Primescan. RMS values from the mesially tilted subgroups showed the lowest RMS deviation values within each intraoral scanner subgroup (*p* < .017). Table 1 presents the percentage of true values achieved by each intraoral scanner at the ± .1 mm tolerance level. When scanning the models with PEEK scan bodies on mesially tilted distal implants, all three intraoral scanners were true more than 70% of the time at the ± .1 mm tolerance.

**Table 1:** Within-tolerance (%) measurements according to implant angulation, scan body materials, and intraoral scanners.

	PEEK			Titanium		
	CS3600	TRIOS3	Primescan	CS3600	TRIOS3	Primescan
Parallel	66.29 [35.21]^a^	67.08 [3.65]^a^	65.17 [5.41]^a^	63.28 [21.99]^b^	81.16 [55.86]^a^	52.2 [3.75]^a^
Mesial	78.53 [4.2]^b^	72.9 [1.64]^b^	72.46 [1.98]^b^	68.85 [5.84]^b^	76.22 [3.33]^a^	67.57 [4.32]^b^
Lingual	57.15 [36.83]^a^	79.49 [1.2]^c^	75.99 [3.82]^c^	36.78 [12.5]^a^	84.15 [37.83]^a^	49.07 [22.64]^a^
p*	.002	< .001	< .001	< .001	.085	.003

Data are presented as median [interquartile range]. Values with the same superscript letters in each column are not significantly different from each other (Mann–Whitney U test followed by Bonferroni correction for multiple comparisons).

*PEEK* polyetheretherketone.

*Kruskal–Wallis test.

The RMS and within-tolerance measurements according to implant angulation and scan body materials are presented in Table 2. Both the PEEK and titanium scan body groups showed better trueness and a higher percentage within tolerance when the distal implant was mesially tilted (*p* < .017). Table 3 presents the RMS and within-tolerance values according to scan body materials. In terms of RMS, scan data from the titanium scan bodies showed better trueness (*p* < .001). However, the within-tolerance value of the titanium scan body group was significantly lower than that of the PEEK group (*p* = .001). The scan data obtained from the different intraoral scanners showed an RMS of 307.9 (136.3), 261.8 (188.4), and 323.9 (145.2) µm and a within-tolerance percentage of 65.79 (30.21), 75.56 (11.34), and 67.11 (17.95) % for the CS3600, TRIOS3, and Primescan, respectively. For both RMS and within-tolerance values, the scan data from the TRIOS3 showed better accuracy than data from the other scanners (*p* < .017), whereas those from CS3600 and Primescan were not significantly different (*p* = .081 for RMS; *p* = .428 for within-tolerance).

**Table 2:** Trueness (root mean square, µm) and within-tolerance (%) measurements according to implant angulation and scan body materials.

	PEEK		Titanium	
	Trueness (RMS, µm)	p*	Trueness (RMS, µm)	p*
Parallel	359.4 [48.3]^b^	< .001	294.1 [110.4]^b^	< .001
Mesial	264.0 [76.5]^a^		150.5 [45.4]^a^	
Lingual	368.5 [70.3]^b^		272.6 [189.2]^b^	

	Within-tolerance (%)	p*	Within-tolerance (%)	p*
Parallel	65.71 [7.03]^a^	< .001	53.90 [24.04]^ab^	.016
Mesial	73.29 [3.80]^b^		69.10 [9.62]^b^	
Lingual	75.99 [17.21]^b^		45.48 [35.75]^a^	

Data are presented as median [interquartile range]. Values with the same superscript letters in each column are not significantly different from each other (Mann–Whitney U test followed by Bonferroni correction for multiple comparisons).

*PEEK* polyetheretherketone, *RMS* root mean square.

*Kruskal–Wallis test.

**Table 3:** Trueness (root mean square, µm) and within-tolerance (%) measurements according to scan body materials.

	PEEK	Titanium	p*
Trueness (RMS, µm)	349.9 [77.3]	222.1 [152.3]	< .001
Within-tolerance (%)	72.37 [11.59]	65.70 [29.20]	.001

Data are presented as median [interquartile range].

*PEEK* polyetheretherketone, *RMS* root mean square.

*Mann–Whitney U test.

## Discussion

Herein, we analyzed the trueness of scan data according to the implant angulation and scan body materials. The results of the deviation analysis between reference and intraoral scan data showed that implant angulation affected the trueness of digital implant impressions (*p* < .001), as did the scan body material (*p* < .001). Therefore, the null hypothesis of this study was rejected.

The results of this study showed that trueness was significantly better when the distal implant was mesially inclined compared to the parallel or lingual inclination, regardless of the intraoral scanner or scan body type. Similarly, Lin et al.^5^ and Zhang et al.^13^ using a partially edentulous mandible model, showed that digital implant impression accuracy was higher in angulated implants. However, in those studies^5,13^, the inclined terminal implants were tilted distally rather than mesially. When the implant at the distal end is mesially tilted in the partially edentulous region, the scan body position emerging supragingally is more mesial than the implant position at the bone crestal level because of the soft tissue thickness. Accordingly, even if the implants are placed at the same mesiodistal point on the bone crestal level, when the distal implant is mesially angulated, the mesiodistal distance above the soft tissue between the mesial and distal implant scan bodies narrows down, thereby reducing the length of the edentulous region between the two scan bodies. The scan data accuracy of plain surfaces is reportedly poor because of intraoral scanner limitations, which make it difficult to stitch the nongeometric surface of the edentulous area^23^. Therefore, a mesial angulation, which shortens the mesiodistal length of the nongeometric edentulous area, might contribute to better implant scan data accuracy.

Several studies using complete edentulous models also reported that implant inclination did not affect intraoral scan data accuracy^6,7,15,17,18^. The present study used models with silicone soft tissue, in contrast to previous studies^6,7,15,17,18^, which used models made of hard materials only^15,18^ or models with silicone soft tissue only in the gingival crest^6,7,13,17^. In this study, we attempted to simulate an actual clinical situation by using entire models covered by silicone soft tissue. The temporal deformation of the elastic soft tissue of the models may reduce the accuracy of the edentulous area stitch. Thus, the decrease in the mesiodistal length of the edentulous area due to the mesial angulation of the implant may improve scan data accuracy. In addition, the use of a model with such an elastic soft tissue could explain the slightly higher deviation values in this study than in previous studies^23,24^. Here, except when the PEEK scan body was scanned with Primescan, no significant difference was found in trueness between the digital implant impressions of the lingually inclined and parallel groups. This finding was consistent with the results of a previous study that found no difference between the accuracy of the scan data of the lingually tilted and vertically placed implants^16^. Since lingual inclination does not reduce the mesiodistal length of the edentulous space between implants compared to the mesial inclination, this may not have influenced the accuracy of scan data.

When comparing the differences according to the scan body materials in this study, the titanium scan body showed a better trueness value than the PEEK scan body. To avoid possible interferences from scan body shape^20,21^, PEEK and titanium scan bodies from the same manufacturer were selected in this study. In this way, we tried to evaluate the independent effect of scan body materials on scan data accuracy. The PEEK and titanium scan bodies used in the present study were similar in shape and dimensions, except that the PEEK scan bodies had one flat face, while the titanium scan bodies had two. However, this difference might have increased the RMS trueness of the titanium scan bodies compared to that of the PEEK scan bodies. Furthermore, the titanium scan bodies had a lower within-tolerance value than the PEEK scan bodies, and the interquartile ranges of the RMS values were wider. In this study, scanning powder was not used in scanning titanium scan bodies according to the manufacturer's instructions; however, the scanning accuracy of shiny metal objects is reportedly worse^25^. Because of the light-reflective surface of titanium scan bodies, the scan results of titanium scan bodies in the present study may be jagged, and this less precise result may be a factor of hesitancy to select titanium scan bodies in clinical practice.

The main limitation of this study is that the in vitro design differs from the oral situation. The distance between the intraoral scanner head and the scan target^26^, ambient light brightness, and color temperature have been reported to influence intraoral scanner accuracy^27^. In this study, the scanning procedures were performed as similar as possible to the clinical situation. However, in the actual oral cavity, scanning the lingual surface using an intraoral scanner is more difficult because of the interference of the tongue and mouth floor. Furthermore, the presence of saliva and humidity may also affect intraoral scan data accuracy^28^. Thus, further clinical studies are needed to confirm the findings of the present study.

Within the limitations of this in vitro study, the trueness of digital implant impressions was influenced by the implant angulation regardless of the intraoral scanner type. In the simulated situation of two implant-supported rehabilitation on three missing posterior teeth, the intraoral scan data showed the highest trueness when the distal terminal implant was placed mesially tilted. The titanium scan body produced significantly better trueness of the acquired scan data compared to the PEEK scan body. However, the percentage within a tolerance of ± .1 mm for the titanium scan body was lower and the interquartile ranges were larger.
